# Fat/Water Separation at 7 T Using a 3D Radial Sequence With Quasi‐Continuous Echo Times

**DOI:** 10.1002/mrm.70323

**Published:** 2026-02-26

**Authors:** Matthias Rohe, Katharina Tkotz, Armin M. Nagel, Stefan Sommer, Max Brockmüller, Florian Knoll, Michael Uder, Nico Egger, Frederik B. Laun, Tobias Wilferth

**Affiliations:** ^1^ Institute of Radiology, University Hospital Erlangen, Friedrich‐Alexander‐Universität Erlangen‐Nürnberg (FAU) Erlangen Germany; ^2^ Division of Medical Physics in Radiology German Cancer Research Centre (DKFZ) Heidelberg Germany; ^3^ Swiss Center for Musculoskeletal Imaging (SCMI) Zurich Switzerland; ^4^ Swiss Innovation Hub, Siemens Healthineers International AG Zurich Switzerland; ^5^ Siemens Healthineers AG Erlangen Germany; ^6^ Department Artificial Intelligence in Biomedical Engineering (AIBE) Friedrich‐Alexander‐Universität Erlangen‐Nürnberg (FAU) Erlangen Germany

**Keywords:** 7 tesla, Dixon MRI, fat/water separation, proton density fat fraction, quasi‐continuous TE, radial sequence

## Abstract

**Purpose:**

To achieve a reliable fat/water separation (FWS) at 7 T using a new 3D radial sequence and reconstruction workflow with quasi‐continuous TE sampling.

**Methods:**

A 3D radial density‐adapted sequence with quasi‐continuous TE sampling was developed for a 7 T whole‐body MRI system. The reconstruction workflow included an off‐resonance correction to reduce chemical shift artifacts. Fat and water signals were separated using a graph cut algorithm, yielding proton density fat fraction maps. The sequence and reconstruction pipeline were tested and validated using phantom measurements and in vivo acquisitions of the lower leg of healthy volunteers.

**Results:**

The proposed sequence and reconstruction pipeline enabled sampling of the fat/water oscillation curve over a range from a minimal mean TE of 0.27 ms to a maximum mean TE of 10.13 ms, with an effective TE increment of 85 μs. The off‐resonant correction significantly reduced chemical shift artifacts in the fat signal. The data points of the fat/water oscillation showed good agreement with a multi‐peak fit function. The quantification of phantom fat percentages was verified with a mean absolute error of 1.5%. The proposed pipeline resulted in consistent interpretation of fat and water signals without any swaps, neither in phantom nor in in vivo measurements.

**Conclusion:**

This study successfully demonstrated the application of a 3D radial sequence with quasi‐continuous TEs for FWS at 7 T. Due to the high sampling rate (effective TE increment below 100 μs), the presented FWS workflow demonstrated high robustness against fat/water swaps, which are typical at ultra‐high field strengths.

## 1 Introduction

Fat/water separation (FWS) is an MRI technique that enables the separation of water and fat signals, both of which are clinically relevant [1, 2, 3, 4]. Additionally, fat and water images can be used to determine the proton density fat fraction (PDFF), which is valuable for assessing diseases characterized by pathologic fat infiltration, including conditions affecting muscle (e.g., sarcopenia or muscular dystrophies) [5, 6, 7, 8, 9, 10] and the liver (e.g., hepatic steatosis) [11, 12].

Dixon proposed the fundamental idea of performing FWS by measuring signals with two different phase conditions between fat and water components [13], which is achieved by acquiring data with different TEs that exhibit, for example, in‐phase and out‐of‐phase states. This basic approach has been subsequently improved via numerous methods, such as the use of multi‐echo measurements [14], multi‐shot acquisitions [15], and the incorporation of *B*_0_ field inhomogeneity, signal decay, and a multi‐peak fat model [16]. To date, FWS has been predominantly applied at field strengths of 1.5 T and 3 T [1, 3, 4, 5, 6] and is rarely applied at 7 T, where the technique is more prone to incorrect classification of fat and water components such as fat/water swaps [17].

The availability and usage of ultra‐high field (UHF) MRI systems with magnetic field strengths of 7 T and above are continuously increasing. These UHF MRI systems offer a higher SNR [18, 19] and better spectral resolution [20] than 1.5 T or 3 T MRI systems, which is especially beneficial for metabolic imaging techniques such as CEST [21] and X‐nuclei MRI [22]. For the quantitative evaluation of such techniques, precise knowledge of the fat fraction is essential [23, 24], requiring a reliable PDFF quantification at 7 T.

Applying FWS at higher field strengths is challenging due to the higher resonance frequency. Dixon‐based FWS is based on the chemical shift of fat, which has a dominant peak at around 3.5 ppm. At 7 T, this chemical shift results in an off‐resonance frequency of slightly above 1 kHz. To use Dixon‐based FWS at this frequency, a TE spacing of less than 0.5 ms is needed to discriminate consecutive in‐phase and out‐of‐phase states, which is not technically feasible with common Cartesian single‐shot sequences at clinically relevant resolution.

Another challenge with UHF MRI systems is *B*_0_ field inhomogeneity, which scales with the main magnetic field strength. This inhomogeneity causes additional phase variation over time, which if not modeled correctly can lead to incorrect assignment of fat and water signals, called fat/water swaps [17]. A higher sampling rate of < 0.5 ms for the fat/water phase oscillation is expected to support more stable FWS, particularly in the presence of *B*_0_ inhomogeneity, which is further investigated in this study.

To achieve such an increased sampling rate, a 3D radial sequence was selected for two reasons. Firstly, it has the advantage that an individualized timing of the spokes can be easily implemented, allowing for an efficient sliding window reconstruction scheme [25] that, together with the golden‐angle distribution of radial spokes [26, 27, 28], enables the reconstruction of image datasets at quasi‐continuous TEs. Here, “quasi‐continuous” means that the effective time increment between reconstructed image datasets is very small (< 100 μs). Secondly, it has the advantage of a short achievable minimum TE, as each readout starts at the k‐space center, which enables sampling of the fat/water phase oscillation very close to the excitation pulse.

Therefore, this study aimed to achieve a reliable FWS at 7 T using a radial pulse sequence with quasi‐continuous TEs.

## 2 Methods

All measurements were performed on a whole‐body 7 T MRI system (MAGNETOM Terra.X; Siemens Healthineers, Forchheim, Germany) equipped with an 80 mT/m @ 200 T/m/s gradient system, using a 28‐channel ^1^H knee coil (Quality Electrodynamics, Mayfield Village, OH, USA).

### 2.1 Quasi‐Continuous Sequence

**FIGURE 1:**
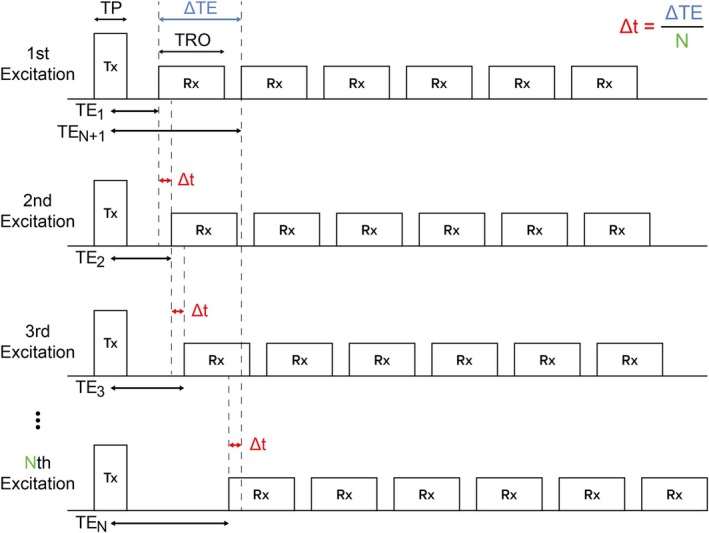
Scheme of the developed density‐adapted 3D radial sequence for a total of *N* excitation pulses (“Tx”). The six gradient readouts (“Rx”) are spaced with a constant ΔTE. For each excitation, all readouts are shifted slightly by Δ*t* = ΔTE/*N*. Therefore, the time interval of several milliseconds has a uniform sampling rate with individualized TEs.

For the proposed FWS workflow, an unbalanced density‐adapted [29] 3D radial research application sequence was developed. The sequence scheme is illustrated in Figure 1. After each excitation pulse (“Tx”), six readouts (“Rx”) separated by the echo spacing ΔTE are acquired. Rewinder gradients are applied after each readout to return the trajectory to the center of k‐space. To acquire data which can be reconstructed at quasi‐continuous TEs, the six readouts are slightly shifted from one excitation pulse to the next by the value Δ*t* = ΔTE/*N*, where *N* is the total number of excitations. Each of these spokes represents a radial projection in k‐space and follows a 3D golden‐angle distribution [26, 27, 28]. Each echo within the same TR uses a separate golden‐angle, so each readout gradient has a different orientation. Due to the time increment Δ*t*, each radial spoke has its own individualized TE spanning from TE_1_ to TE_6N_, which is then discretized to match the gradient raster time of 10 μs.

The following parameters were used for the measurements (Figure 1): nominal flip angle FA = 3°, TR = 15 ms, rectangular excitation pulse with TP = 0.1 ms, TE_1_ = 0.1 ms (time from the center of the excitation pulse to the beginning of the readout), and 20 000 excitations, resulting in 120 000 total spokes. Within one TR, six monopolar readouts were performed with a readout time TRO = 0.42 ms and ΔTE = 1.7 ms. The measurements had a nominal isotropic spatial resolution of (1.0 mm)^3^. The total acquisition time was 5 min.

### 2.2 Reconstruction

Image datasets were reconstructed using an in‐house pipeline implemented in MATLAB (version R2022b; The MathWorks, Natick, MA, USA). A Hamming filter was applied to the k‐space data to reduce Gibbs ringing and improve the SNR [30, 31]. The k‐space data were density compensated, and the corresponding image datasets were obtained by applying a nonuniform fast Fourier transform [32].

To correct for gradient imperfections, the actual k‐space trajectory was measured using the thin‐slice method described by Duyn et al. [33]. A spherical phantom was positioned at the scanner isocenter for the trajectory measurements. For each of the three physical gradient axes, trajectories were measured in two off‐center slice positions (±40 mm relative to isocenter), as required by the thin‐slice method. The measurement covered the entire radial readout waveform, including ramp‐up, flat‐top, and density‐adapted segments. Trajectories were measured separately for the three physical gradient axes, and arbitrary radial spoke orientations were calculated by linear superposition. The measurement was performed for a single echo, whereas the quasi‐continuous acquisition employs multiple echoes. The measured k‐space trajectory was then used during image reconstruction, thereby compensating for gradient inaccuracies. Figure S1 shows the measured gradient waveforms in comparison to the nominal gradients and the resulting impact on the images.

The golden‐angle distribution allows image reconstruction with any subset of the spokes. Since the gradient readouts have individualized TEs, reconstruction from a subset of the acquired spokes yields an image dataset spanning a range of TEs rather than a single TE. For each reconstructed image dataset, the effective TE was defined as the mean TE of all spokes included in that window.

To obtain quasi‐continuous TEs, a sliding window scheme [25] was used to process all spokes. A fixed‐width window of 4000 spokes was moved across the full golden‐angle dataset of 120 000 spokes with a defined step size of 1000 spokes, yielding a total of 117 image datasets. This window size corresponds to a TE range of 340 μs, used for all image datasets, with an effective TE increment of 85 μs between consecutive datasets. Because the window shifts by 25% of its width, consecutive image datasets share 75% of their spokes, ensuring smooth temporal evolution of contrast while generating a large number of distinct mean TEs to sample the fat/water oscillation with a high temporal resolution. Notably, this method does not reduce physical echo spacing within a TR; instead, it generates a finely sampled effective TE grid during multi‐TR acquisitions.

In this study, phantom measurements were reconstructed to a matrix of size (150)^3^, and in vivo measurements were reconstructed to a matrix of size (200)^3^, corresponding to FOVs of (150 mm)^3^ and (200 mm)^3^, respectively.

**FIGURE 2:**
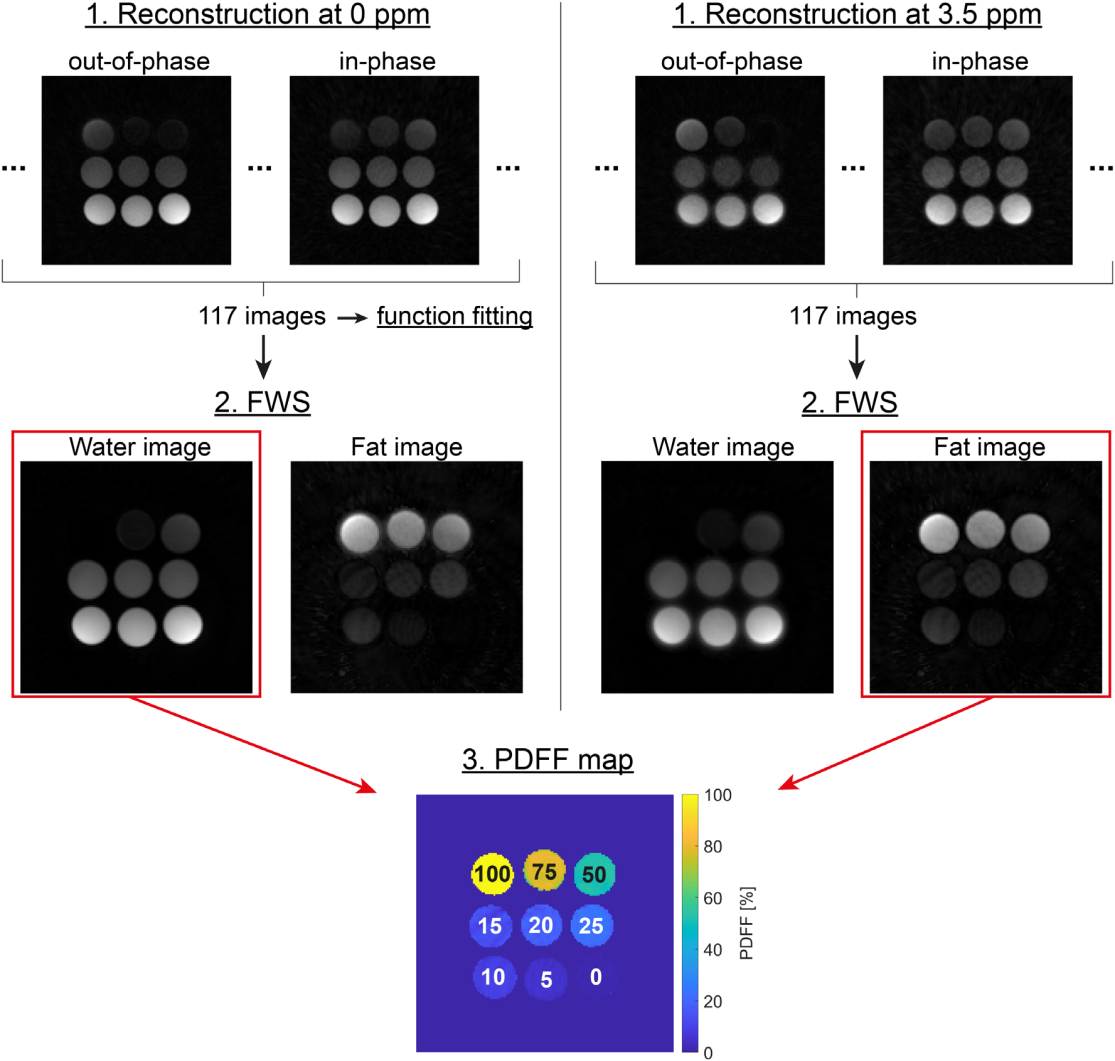
Reconstruction workflow. Each measurement was reconstructed twice: once at the water frequency (left) and once by applying an off‐resonance correction in k‐space to shift the effective resonance frequency to the main fat peak (3.5 ppm for the phantom) (right). The magnitude images at 0 ppm are used as data points for fitting the multi‐peak function to the oscillation (Figure 3). Next, the 117 resulting image datasets are used for FWS applied to each reconstruction. The water image without off‐resonance correction and the fat image with off‐resonance correction were used to calculate the PDFF. The ground truth values of the phantom vials are also shown in the PDFF map.

Image datasets can be reconstructed to account for different off‐resonances, thereby optimizing the quality of the fat image, as chemical shift produces isotropic blurring in radial trajectories. For each measurement, the image datasets were reconstructed once with a linear phase shift of the dominant fat peak (phantom: $\omega_{\mathrm{off}}=3.5$ ppm, in vivo: $\omega_{\mathrm{off}}=3.2$ ppm) to obtain an optimized fat image, and once without any shift to obtain an optimal water image. The workflow is shown in Figure 2. This off‐resonant reconstruction was implemented by adding a linear phase shift to each complex k‐space value dependent on its respective sampling time t:

(1) $$k_{\text{shift}}\left(t\right)=k_{\text{original}}\left(t\right)\cdot e^{i\omega_{\mathrm{off}}t}.$$

here, $k_{\text{shift}}\left(t\right)$ is the time‐dependent phase‐shifted k‐space value, $k_{\text{original}}\left(t\right)$ the time‐dependent original k‐space value, and $\omega_{\mathrm{off}}$ is the dominant off‐resonance angular frequency of fat.

The total image reconstruction time (excluding FWS) for one measurement was approximately 1.5 h for the phantom data and 2.5 h for the in vivo data. All reconstructions were performed on a workstation equipped with an Intel i9‐14900 CPU and 192 GB RAM, using CPU‐based parallelization and without GPU acceleration.

### 2.3 Phantom and Volunteers

The phantom [17, 34, 35] consisted of nine vials containing emulsions of peanut oil and agarose (3% w/v) with varying volume fat fractions (VFFs): 0%, 5%, 10%, 15%, 20%, 25%, 50%, 75%, and 100%. Phantom measurements were performed in the isocenter of the scanner.

For the in vivo images, the right calves of two healthy female volunteers (21 and 24 years old) were scanned. Written informed consent was obtained from these volunteers, and their examination was approved by the local ethics review board. Subject measurements were acquired with the calf positioned slightly off‐isocenter, reflecting the practical positioning of the dedicated knee coil.

### 2.4 Fit Function

Regions of interest (ROIs) were manually drawn on a single slice of the magnitude image datasets reconstructed at 0 ppm, leaving a margin around the target to minimize partial volume effects. For the phantom datasets, a circular ROI was drawn in each vial. For the in vivo datasets, three ROIs were drawn: one covering the muscle tissue, one in the tibial bone marrow, and one in the subcutaneous fat above the tibia.

To test the agreement of the acquired data points s(t) (mean value over ROIs), a multi‐peak function was used to fit the oscillation curves [1, 16]:

(2) $$\left|s\left(t\right)\right|=\left|\left(\rho_{w}+\rho_{f}\;c\left(t\right)\;e^{i2\pi \Delta \omega_{f}t}\right)\cdot e^{-R_{2}^{*}t}\right|\;\text{with}\;c\left(t\right)=\sum_{p=1}^{P}\alpha_{p}\;e^{i2\pi \Delta \omega_{p}t}.$$

here, $\rho_{w}$ and $\rho_{f}$ are the amplitudes of the water and fat components, respectively; $\alpha_{p}$ the relative amplitude of the peaks in the multi‐peak fat model; $\Delta \omega_{p}$ is the angular frequency shift (with respect to water) of fat peak p; $\Delta \omega_{f}$ is the temperature‐dependent frequency shift; and $R_{2}^{*}$ is the transverse relaxation rate of the signal. The fit parameters were $\rho_{w},\rho_{f},\Delta \omega_{f}\;\text{and}\;R_{2}^{*}$. The nine‐peak phantom and seven‐peak in vivo fat models were adopted from previously published spectral measurements [34, 36].

To quantify the agreement of the resulting fit ($s_{\mathrm{fit}}$) to the measured data ($s_{\text{data}}$), the normalized root mean square error (NRMSE) was calculated:

(3) $$\text{NRMSE}=\frac{\sqrt{\frac{1}{n}\sum_{i=1}^{n}{\left(s_{\text{data},i}-s_{\mathrm{fit},i}\right)}^{2}}}{s_{\text{data},\max}-s_{\text{data},\min}}.$$

here, n is the total number of sample points, $s_{\text{data},\max}$ is the maximum value, and $s_{\text{data},\min}$ is the minimum value of the measured data.

### 2.5 Separation Algorithm and PDFF Calculation

A 2D graph cut algorithm [37] was used to determine the voxel‐wise FWS. A nine‐peak fat model [34] was used for the phantom datasets, and a seven‐peak fat model [36] was used for the in vivo datasets. The algorithm was applied twice for each measurement: once with a subset of slices from the image dataset reconstructed at the water frequency, and once with a subset of slices from the image dataset reconstructed at the main fat frequency. The total runtime of the FWS algorithm for one slice was approximately 20 min for phantom data and 30 min for the in vivo data (same hardware specifications as above).

To calculate a PDFF map from the resulting fat image F (with off‐resonance correction) and water image W (without off‐resonance correction), the following equation was used:

(4) $$\text{PDFF}=\frac{\left|F\right|}{\left|F\right|+\left|W\right|}.$$

For quantification, the mean estimated PDFF in phantom ROIs was compared to the ground truth VFF. The mean absolute error (MAE) was calculated from these two values using the following equation:

(5) $$\mathrm{MAE}=\frac{1}{9}\sum_{i=1}^{9}\left|{\mathrm{VFF}}_{i}-{\text{PDFF}}_{i}\right|.$$

### 2.6 Cartesian Reference Sequence

To assess and compare the results obtained with quasi‐continuous TE sampling, phantom and in vivo measurements were also acquired using a conventional Cartesian six‐point Dixon sequence, which was optimized for 7 T calf applications [17]. It uses a 3D FLASH volumetric interpolated breath‐hold examination gradient echo sequence with six monopolar readouts separated by a ΔTE of 2.3 ms. The following parameters were used for the measurements [17]: nominal FA = 3°, TR = 21 ms, FOV = 200 mm, bandwidth BW = 900 Hz/Px, and a voxel size of 1.0 × 1.0 × 5 mm^3^. The phase encoding direction was L−R. In total, 16 slices were acquired, with an acquisition time of 1:06 min.

### 2.7 Assessment of *B*_0_ Inhomogeneity Robustness

To evaluate the robustness of our quasi‐continuous TE sampling scheme against *B*_0_ inhomogeneity, it was compared with the conventional six‐point Dixon sequence, acquiring datasets under different shim conditions. First, a reference scan was performed using a standard shimming protocol. Next, the shim currents were manually adjusted to introduce a field inhomogeneity along the *x*‐axis. Several measurements with increasing strength of this *B*_0_ gradient were performed to assess the sensitivity of each acquisition method to fat/water swaps induced by *B*_0_ inhomogeneity.

## 3 Results

Since each measurement was reconstructed twice, the results were two sets with 117 image datasets each. After FWS with the graph cut algorithm (Figure 2), the image datasets reconstructed without an off‐resonance correction shift showed water images of good quality and fat images with blurred edges due to chemical shift. In contrast, the reconstruction at 3.5 ppm produced a water image with visible chemical shift artifacts, but a fat image with significantly improved sharpness. Figure S2 provides a quantitative assessment of the impact of the off‐resonance correction on the fat image.

**FIGURE 3:**
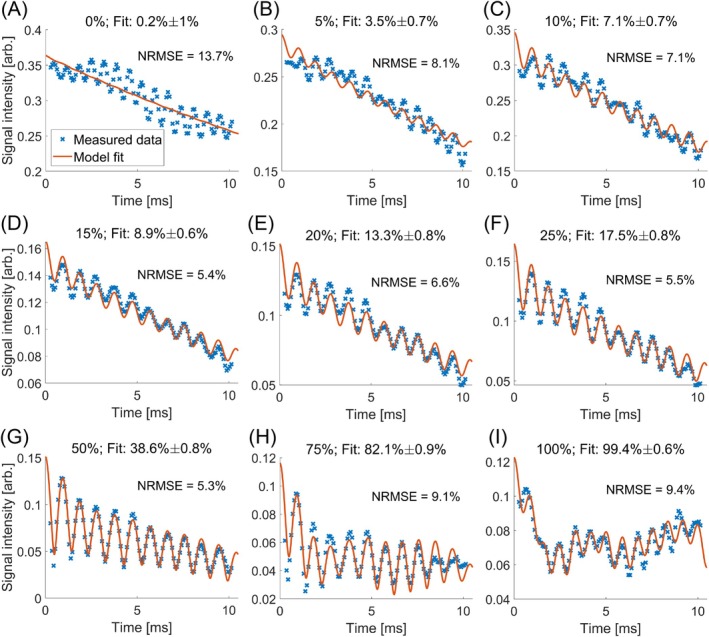
Plots of the measured fat/water shift oscillations (blue *x*‐markers) fitted with a multi‐peak fat signal model (orange solid line) obtained from the 117 phantom images. The resulting fat fraction and respective fit parameter uncertainty are reported at the top of each plot. The signal intensities were acquired with a phantom containing nine vials with different proportions of peanut oil and agarose (3% w/v): 0% (A), 5% (B), 10% (C), 15% (D), 20% (E), 25% (F), 50% (G), 75% (H), and 100% (I). The high sampling rate enables a reliable matching of the fitted model curve and the quasi‐continuous data points. The NRMSE was highest for VFF_0_ (13.7%). The fat fraction was consistently underestimated for VFFs from 5% to 50%, with relative errors ranging from approximately 23% (VFF_50_) to about 41% (VFF_15_). Note that the signal intensity axes differ across the plots.

Figure 3 shows the ROI‐averaged signal intensity of each vial of the phantom across all 117 TEs, to which the multi‐peak function was fitted. Due to the high number of sampling points, reliable fitting of the oscillations was possible. The fitted curves showed good agreement with the measured data for VFFs > 0% (NRMSE < 10%). For VFF_0_ (0% ground truth), the data showed some oscillation and spread that are not reproduced by the fitted model (NRMSE = 13.7%), although the resulting fat fraction matched the VFF. For lower VFFs of 5%–50%, the fitted model underestimated the fat fraction by up to a relative error of approximately 41%. For VFF_75_, the fat fraction was slightly overestimated with a relative error of 9.5%. For VFF_100_, the fitted model could capture the intensity variations due to the different off‐resonant frequencies.

**FIGURE 4:**
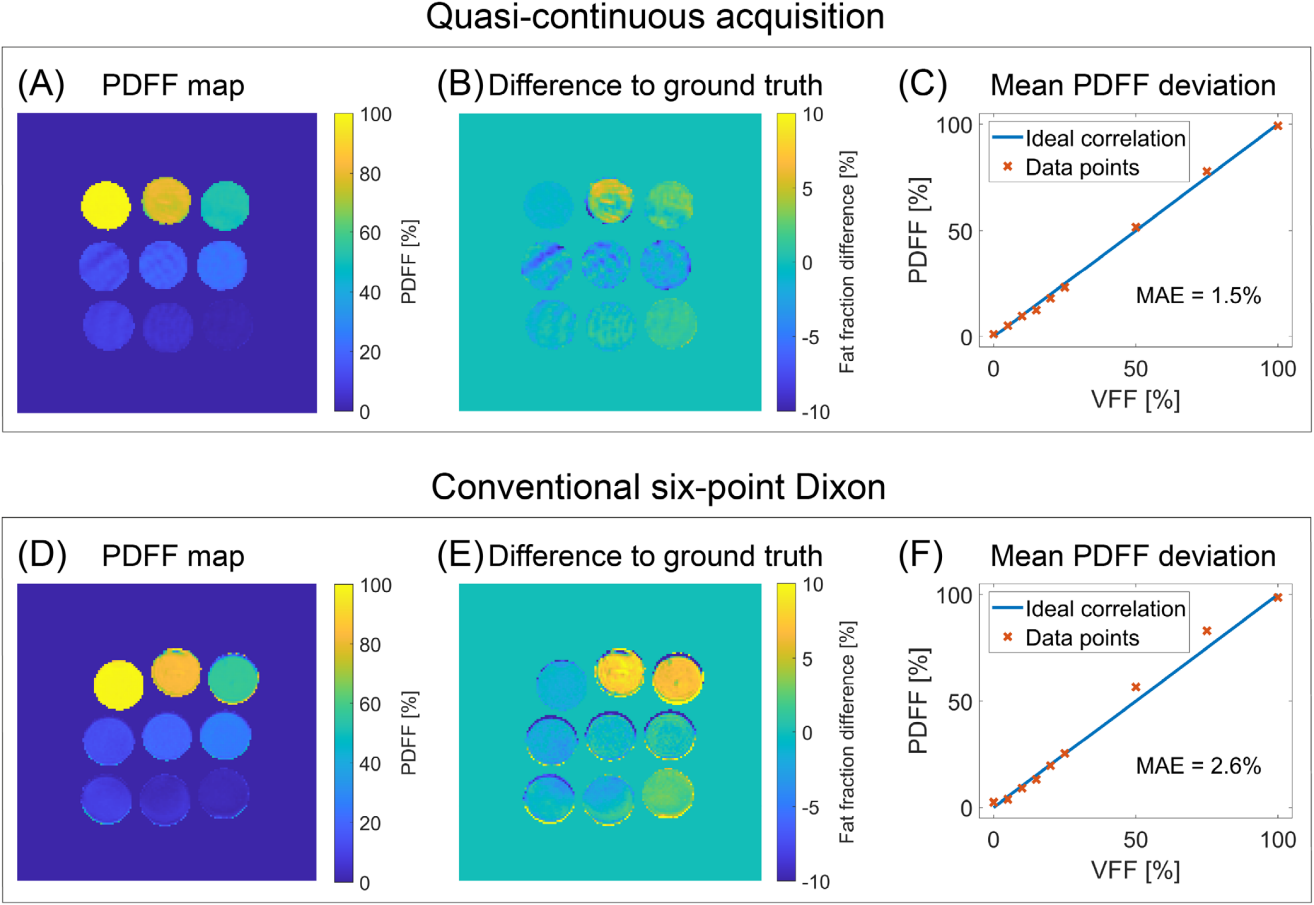
Results of FWS with the graph cut algorithm applied to the phantom data for the quasi‐continuous and conventional Cartesian six‐point Dixon acquisitions. (A and D) The calculated PDFF map. (B and E) The difference between the resulting PDFF and the ground truth. The greatest deviation occurred for the 50% and 75% vials with the conventional sequence. (C and F) The nine mean PDFF values from each vial were compared with the ideal correlation, yielding MAEs of 1.5% and 2.6%, respectively. The spatial heterogeneity observed for the quasi‐continuous acquisition likely originates from temporally averaged phase evolution introduced by the sliding window reconstruction, which affects the resulting fat image and propagates into the PDFF maps. The Cartesian Dixon acquisition uses fixed echo times and therefore does not exhibit comparable heterogeneity.

Figure 4 shows the FWS results for the phantom with the graph cut algorithm for the quasi‐continuous and conventional six‐point Dixon acquisitions. The PDFF maps show homogeneous values within the vials with both sequences. However, the quantification is more accurate with the quasi‐continuous acquisition (MAE: 1.5% vs. 2.6%). This difference arises primarily due to the six‐point Dixon sequence overestimating the 50% and 75% vials by approximately 7% and 8%, respectively.

**FIGURE 5:**
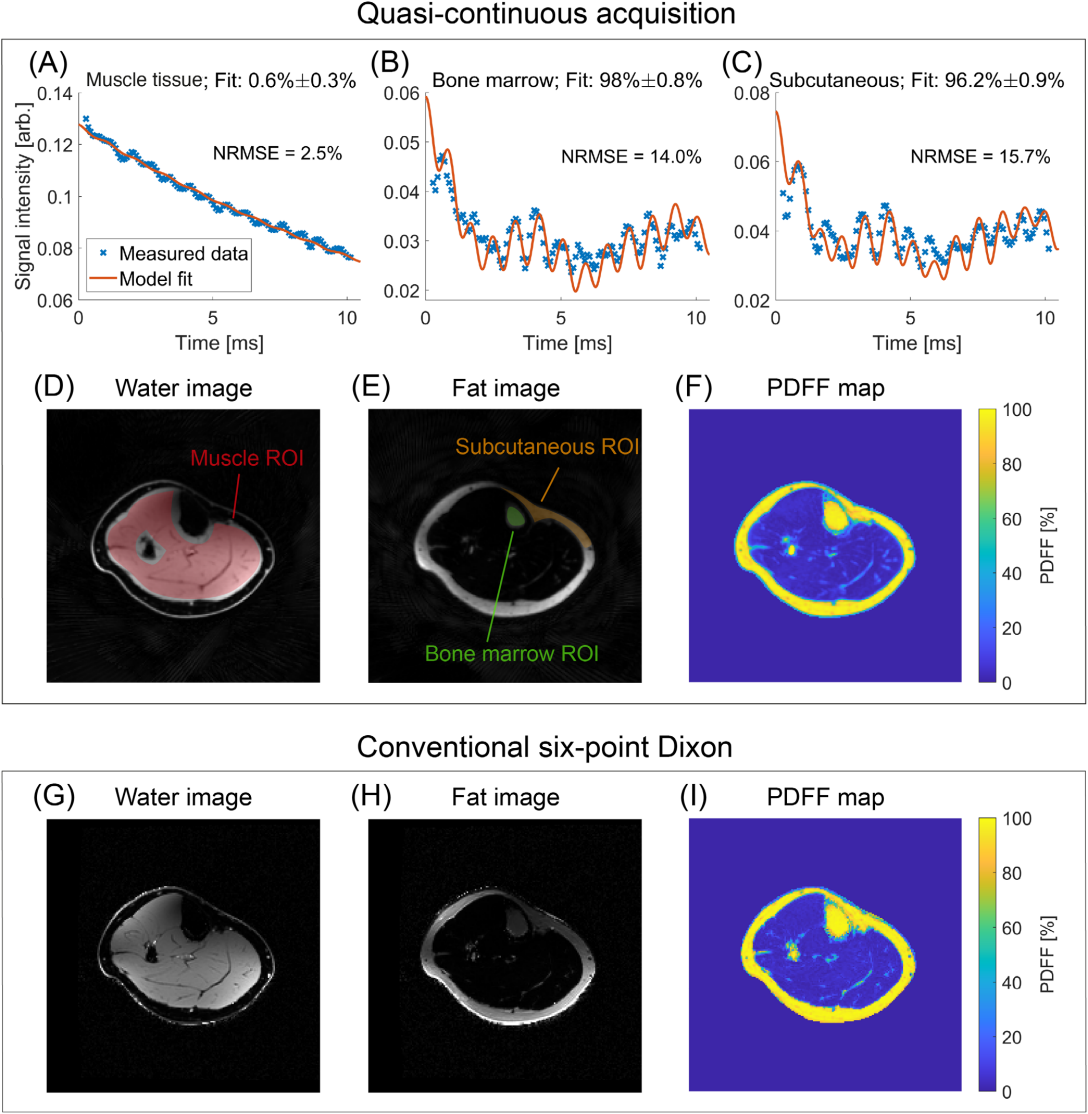
Results of the in vivo measurements of a healthy 21‐year‐old female volunteer (Subject 1). (A–C) The mean signal intensity (blue *x*‐markers) and the corresponding fit (orange solid line) in muscle tissue (A), subcutaneous fat (B), and bone marrow (C). (D–I) FWS results from the graph cut algorithm for both acquisitions. The water image (D) and fat image (E) from the quasi‐continuous acquisition are overlaid with the three ROIs for the three tissue groups in the plots on the upper row. The PDFF maps (F and I) show similar PDFF values inside the muscle and fatty tissue.

Figure 5 shows the in vivo results for Subject 1. For muscle, the temporal signal intensity evolution follows a steady decay with minor oscillations, which are not correctly modeled by the fit function, resulting in an estimated fat fraction of 0.6%. For bone marrow and subcutaneous fat, the model fits had higher NRMSE values of 14.0% and 15.7%, respectively, and the fitted fat fractions are close to 100% for both ROIs.

In the water images produced by FWS with the graph cut algorithm, individual muscle structures and blood vessels can be delineated for both acquisition methods. In the fat images, the bone marrow, subcutaneous fat, and also some small structures in the muscle can be observed. The quasi‐continuous and conventional acquisitions yielded water and fat images of similar quality following FWS, although the latter appears to yield higher effective resolution than the former.

Lastly, the PDFF maps were calculated, which look very similar for both acquisitions. Notably, no fat/water swaps were observed in any of the resulting images.

**FIGURE 6:**
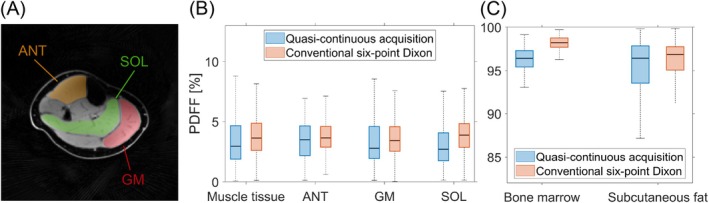
Quantitative evaluation of PDFF in Subject 1 comparing the quasi‐continuous acquisition with the conventional six‐point Dixon sequence. (A) Water image obtained with the quasi‐continuous acquisition showing three further ROIs used for analysis: Anterior segment (ANT), gastrocnemius medialis (GM), and soleus (SOL). (B and C) Box plots of PDFF values across all six ROIs in total (muscle tissue, bone marrow, and subcutaneous fat as defined in Figure 5) for both acquisition types. Overall, PDFF values obtained with the quasi‐continuous acquisition are similar to those from the conventional six‐point Dixon sequence across all tissue ROIs, with slightly lower median values and increased variability observed for the quasi‐continuous acquisition.

Figure 6 shows a quantitative comparison of PDFF values for Subject 1 between the two acquisition types across six ROIs. Overall, PDFF values are comparable between both sequences for muscle and fatty tissues. For the combined muscle ROI, the median PDFF was 3.0% for the quasi‐continuous acquisition and 3.6% for the Cartesian reference. This trend of slightly lower PDFF values with the quasi‐continuous approach was consistently observed across the individual muscle ROIs (ANT, GM, and SOL), with absolute differences of the median PDFF ranging from 0.2% (ANT) to 1.2% (SOL). Similarly, in fatty tissues, median PDFF values were higher for the Cartesian acquisition, with absolute differences of 1.8% in bone marrow and 0.4% in subcutaneous fat.

Across all ROIs, the quasi‐continuous acquisition exhibited increased variability, reflected by larger interquartile ranges, which were up to 1.7 percentage points higher than those obtained with the Cartesian sequence.

**FIGURE 7:**
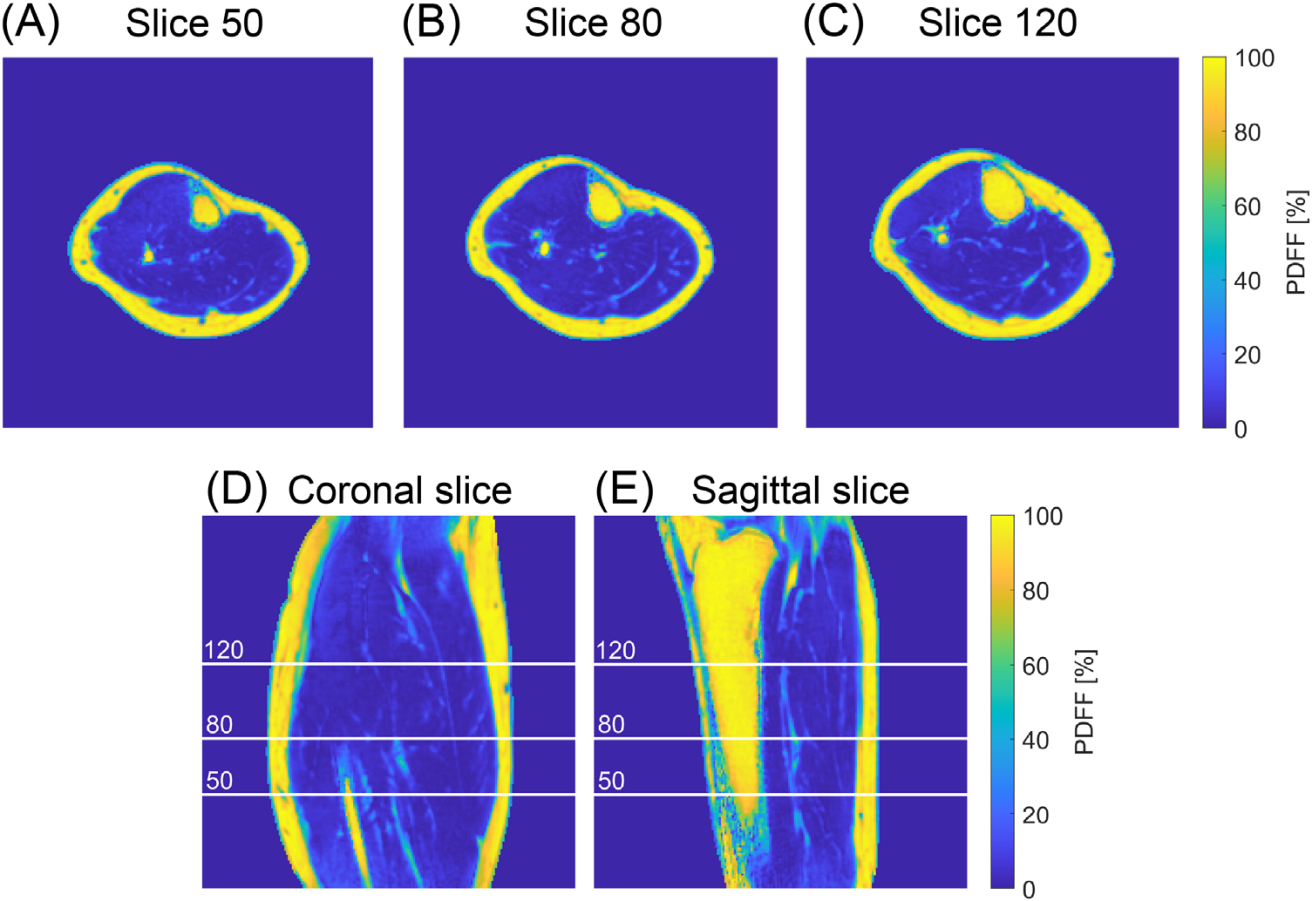
In vivo FWS results of Subject 1 across multiple slices and planes. The top row (A–C) shows three transverse slices, where slice 80 is the same as shown in Figure 5. The bottom row (D and E) shows a coronal and sagittal slice, respectively (with indicated lines for the transverse slices). The quantification of the PDFF is robust and consistent for all five images. Only in the bottom‐left of the sagittal slice did the reconstructed images have a very low SNR, which affected FWS.

Figure 7 shows the in vivo FWS results for Subject 1 across multiple transverse slices and a coronal and sagittal slice as well. Quantification of muscle tissue, bone marrow, and subcutaneous fat performed well for all depicted slices. The transverse slices show a similar quality in fine structures present in the muscle and subcutaneous fat. In the sagittal slice, the bottom region showed very low signal, leading to a less reliable PDFF interpretation. For comparison, Figure S3 presents three sagittal slices from a phantom measurement.

**FIGURE 8:**
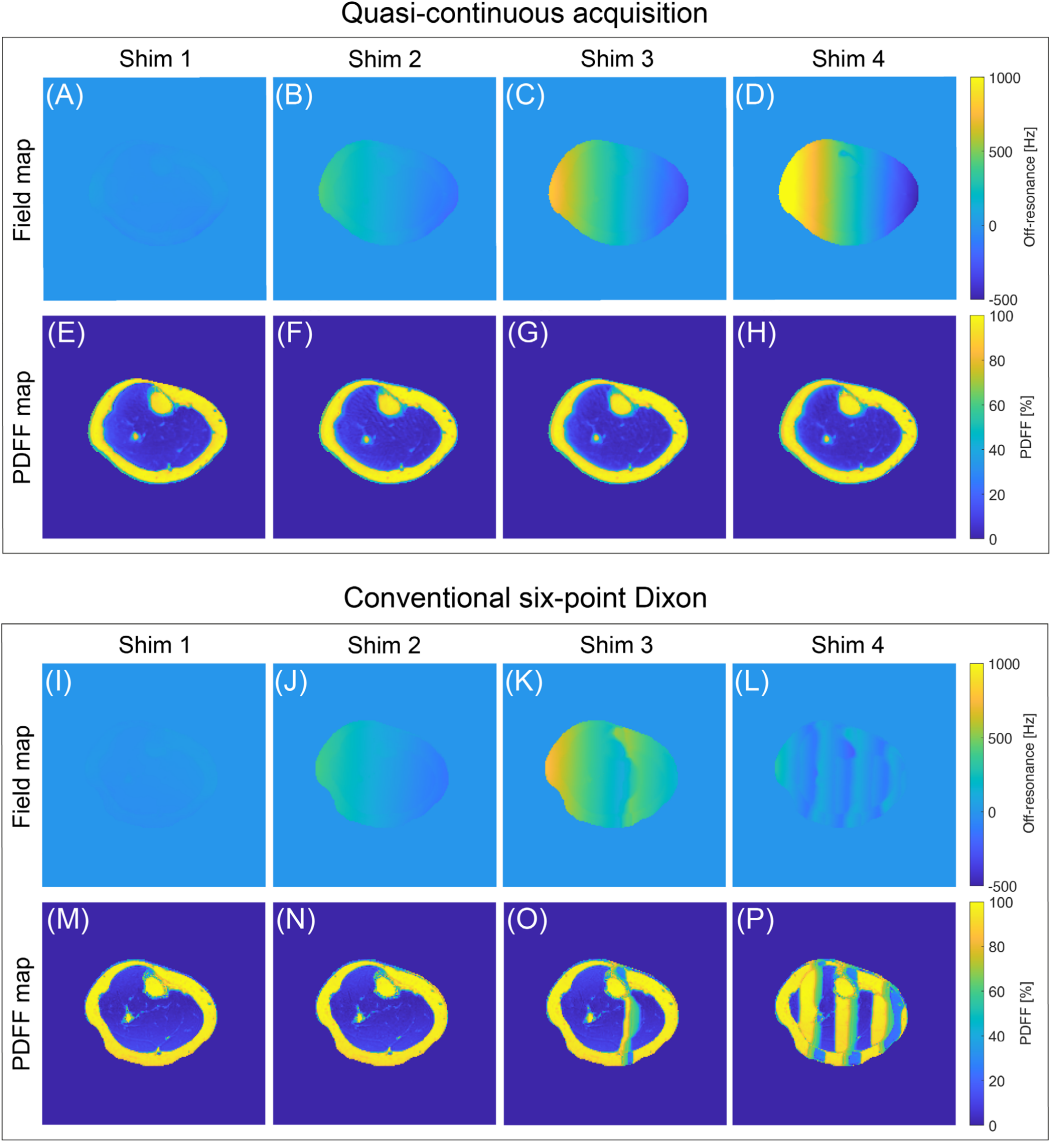
FWS results for Subject 2 under different shim conditions for both the quasi‐continuous and the conventional six‐point Dixon sequences. The shim currents were changed manually to generate increasingly strong *B*_0_ *x*‐gradients for shims 2–4. Field maps obtained with the quasi‐continuous acquisition (A–D) model this behavior correctly, whereas the field maps from the conventional acquisition (I–L) struggle with shims 3 and 4. That behavior propagates into the PDFF maps (E–H and M–P), where the conventional acquisition results in fat/water swaps in the calf. Notably, the quasi‐continuous acquisition yields images that are almost identical across all shim conditions.

Figure 8 shows the FWS results for Subject 2 across different shim conditions, comparing the quasi‐continuous and conventional six‐point Dixon sequences. The estimated field maps obtained with the graph cut algorithm highlight the difference in robustness between the two acquisitions in the presence of appreciable *B*_0_ inhomogeneity. The shim currents were modified to introduce an artificial *B*_0_ inhomogeneity in the *x*‐direction. Four shim sets were defined, of which sets 2–4 introduced progressively stronger *B*_0_ *x*‐gradients. With the conventional acquisition, the FWS algorithm could not model the field correctly after shim 2. This inaccuracy propagates into the PDFF maps, where a vertical fat/water swap appears for shim 3. For shim 4, multiple swaps appear in the calf. With the quasi‐continuous acquisition, the field maps exhibit the expected behavior of an increasing *B*_0_ *x*‐gradient across the different shim sets. By modeling this appreciable inhomogeneity correctly, FWS yields almost identical PDFF maps for every shim without any fat/water swaps. The median PDFF for muscle decreases slightly from 5.6% with shim 1 and 4.5% with shim 4. A corresponding phantom experiment is shown in Figure S4, where a fat/water swap appears for the Cartesian acquisition but not for the proposed quasi‐continuous method under similar *B_0_* inhomogeneity.

## 4 Discussion

This study successfully demonstrated that the proposed 3D radial sequence with quasi‐continuous TE sampling circumvents the technical echo spacing limitations of FWS, which become very prominent at 7 T due to the high relative oscillation frequency of fat and water. One advantage of the proposed radial sequence was the absence of fat/water swaps, even in the presence of appreciable *B*_0_ inhomogeneity, likely due to the increased number of sampling points.

Unlike earlier approaches, which sampled k‐space fully for each TE [38], the proposed radial sequence samples spokes with distinct TEs via a time shift Δ*t* after each excitation pulse. Since the spokes have distinct timing and a sliding window scheme was used for reconstruction, the fat/water shift could be sampled with 117 data points within a few milliseconds and an effective TE increment of 85 μs. Notably, however, the achieved effective TE increment of 85 μs does not represent a required value for FWS at 7 T but rather is a consequence of using a sliding window for reconstruction.

The fat fraction could be estimated with acceptable accuracy for the phantom (Figure 3) and right calves of volunteers (Figure 5, top row) by fitting a multi‐peak model to the magnitude data. The fit precision (NRMSE) ranged from 2.5% to 15.7%, suggesting limitations in the approach to fitting the mean signal intensity oscillation with the defined multi‐peak model. Additionally, relative deviations of up to 41% were observed with this method. These observations are consistent with the established fact that fat can be quantified more robustly and accurately from complex MR signals than from magnitude data [37, 39].

For the phantom, quasi‐continuous fat quantification with the graph cut algorithm which uses the complex MR signal showed greater accuracy (MAE = 1.5%). There were slight underestimations of a few percentage points for the ground truth VFF_15_ and VFF_20_ and an overestimation by 2.5% for VFF_75_ while the other values were almost in perfect agreement. These deviations might be explained by differences between the fat model and the actual off‐resonance frequencies. Additionally, although the fitted T2* relaxation times vary between the individual fat and water signals, the algorithm considered only a single relaxation time per voxel. The conventional six‐point Dixon acquisition performed worse than the proposed quasi‐continuous acquisition, notably overestimating fat fractions for VFF_50_ and VFF_75_, resulting in an MAE of 2.6%, which is 1.1% higher than that of the proposed method.

For the in vivo data, the FWS obtained with the graph cut algorithm was highly similar in terms of quality and fat quantification with both acquisitions. The quasi‐continuous acquisition yielded slightly lower PDFF values and increased variability compared with the conventional six‐point Dixon sequence across all tissue ROIs. Water and fat images had slightly higher effective in‐plane resolutions with the conventional sequence, which can be explained by the nonuniform and spherical k‐space coverage of the 3D radial trajectory that produces a broader point spread function than a cuboid Cartesian acquisition [40, 41]. In addition, the applied Hamming filter in the radial image reconstruction further smooths the data and reduces spatial resolution [30]. Moreover, the radial datasets were highly undersampled relative to the Nyquist criterion. A future goal would be to match this effective resolution with our proposed quasi‐continuous acquisition by increasing the nominal resolution.

Notably, the PDFF maps across multiple transverse slices and in multiplanar form demonstrate the spatial robustness of the proposed quasi‐continuous acquisition.

To further test the robustness of the quantification with respect to *B*_0_ inhomogeneity, in vivo measurements were taken under varying shim conditions. The shim was manipulated so that the resulting *B*_0_ field exhibited a non‐vanishing *x*‐gradient of varying strengths. For zero or small *x*‐gradients, both the quasi‐continuous and conventional acquisitions performed similarly well in estimating the field map and quantifying the PDFF. For larger *x*‐gradients, fat/water swaps were clearly visible in the PDFF maps from the conventional six‐point Dixon acquisition. In contrast, our proposed quasi‐continuous acquisition showed no swaps and consistent quantification across all tested shim conditions. Thus, appreciable *B*_0_ inhomogeneity, which is especially pronounced at UHF, had only a negligible impact on fat quantification with the proposed sequence. Therefore, the proposed quasi‐continuous acquisition could also serve as a robust *B*_0_ mapping tool in the presence of large field inhomogeneity.

The demonstrated robustness against *B*_0_ inhomogeneity may also have important implications for liver fat quantification at UHF. Future studies will be required to validate the approach in abdominal applications, especially considering motion, larger FOV demands, and stronger susceptibility gradients.

An off‐resonance correction was introduced during image reconstruction, enabling sharper fat images with reduced blurring from chemical shift artifacts. Since this study focused on the feasibility of a radial sequence with quasi‐continuous TE sampling for FWS, such a simple off‐resonant reconstruction was chosen. While this alone had a positive impact on the fat images and the resulting quantification, the artifacts did not completely disappear, which could be addressed using more sophisticated reconstruction methods [42, 43, 44]. Another important limitation of the proposed reconstruction approach is the assumption of a global phase shift due to fat off‐resonance without accounting for voxel‐wise *B*_0_ inhomogeneity, which could be implemented in a further step [43].

The golden‐angle distribution of the radial spokes used in our proposed approach exacerbates gradient inaccuracies and eddy currents due to the large, rapid gradient change [28]. These gradient non‐linearities were corrected using an established method that tracks the actual k‐space trajectory [33]. However, using more sophisticated approaches, such as mapping the magnetic field with a field probe [45, 46] or incorporating the behavior of the gradient system with a gradient impulse response function [47, 48, 49], could further improve image quality and correction accuracy.

Another possible improvement is to shorten the acquisition and post‐processing time. The acquisition time of 5:00 min can be further reduced by optimizing the number of spokes and, therefore, also the number of resulting TEs. The goal would be to approach the scan duration of the corresponding Cartesian acquisition (just over 1 min). It is important to note, however, that unlike Cartesian imaging with anisotropic voxels (as used in this study for comparison), the 3D radial readout inherently yields isotropic voxels, providing high‐quality multiplanar reformations. In addition, the total time for reconstruction and FWS of approximately 2 h for the phantom data and 3 h for the in vivo data could also be further optimized by using more efficient code implementations and by employing more powerful hardware (e.g., GPU‐accelerated systems or dedicated servers).

## 5 Conclusion

This study successfully implemented a 3D radial sequence with quasi‐continuous TEs for FWS at 7 T and tested it with phantom and in vivo data. The presented measurement and workflow enabled an effective TE increment of 85 μs, thereby enabling a high sampling rate of the fat/water phase oscillation, yielding consistent and reliable interpretation of the fat and water signal, even in the presence of appreciable *B*_0_ inhomogeneity. Future development will focus on refining image quality and further improving the efficiency of the acquisition and reconstruction process to enable clinical deployment.

## Funding

This work was supported by the Interdisciplinary Center for Clinical Research (IZKF) at the University Hospital of the University of Erlangen‐Nuremberg (Junior Project J123) and by the German Research Foundation (DFG) (project 500888779/RU5534).

## Conflicts of Interest

Stefan Sommer is an employee of Siemens Healthineers International AG. Max Brockmüller is an employee of Siemens Healthineers AG. Other authors declare no conflicts of interest.

## Supporting information


**Figure S1:** Results of the measured gradient trajectory using the thin‐slice method and its impact on the images. (A–C) Measured gradient waveforms in comparison with the nominal gradient waveform for *x*‐, *y*‐, and *z*‐direction. Deviations are most pronounced at the beginning of the readout and immediately after the ramp‐up (zoomed‐in regions). The measured waveforms were incorporated into the reconstruction pipeline. (D–G) Water and fat images obtained with image data sets using the nominal gradient waveform (D and E) and the measured gradient waveform (F and G). Incorporating the measured trajectory substantially improves image quality for both contrast components. The blurred edges, particularly on the left side of the images, are effectively corrected, resulting in sharper depiction of the entire calf.
**Figure S2:** Quantitative assessment of the off‐resonance correction on the fat images. (A) Fat image which was reconstructed at 0 ppm (without off‐resonance correction). (B) Fat image which was reconstructed at 3.5 ppm (with off‐resonance correction). In both images, a red horizontal line indicates the location of the intensity profiles shown in (C). The normalized line profiles demonstrate that the fat image with off‐resonance correction exhibits a much steeper signal drop, approaching zero between edges, whereas the uncorrected image retains elevated intensity levels, indicating blurring and signal spreading. This confirms that the off‐resonance correction improves the sharpness of the fat images.
**Figure S3:** Phantom FWS results across three sagittal planes. The top row (A–C) shows the PDFF maps of the three sagittal slices, with the respective VFF ground truth values indicated inside the vials. The bottom row (D–F) displays the difference between the estimated PDFF maps and the ground truth. No large deviations appear in the sagittal plane, demonstrating the spatial robustness of the proposed method for the phantom data. The deviations that are visible are evenly distributed within the corresponding vials and are consistent with the transverse results shown in Figure 4. This spatial heterogeneity likely originates from temporally averaged phase evolution introduced by the sliding window reconstruction, which affect the resulting fat image and propagate into the PDFF maps.
**Figure S4:** Phantom FWS results under two different shim conditions for both the quasi‐continuous (A–D) and the conventional six‐point Dixon sequences (E–H). The top row shows the *B*_0_ field maps and the bottom row shows the corresponding PDFF maps. The columns labeled “initial shim” display the results after a standard shimming protocol. As expected, the field maps (A and E) exhibit no substantial off‐resonances, and the PDFF estimates (C and G) agree well with the ground truth values. The columns labeled “added *B*_0_ *x*‐gradient” show the results after manually adjusting the shim currents to introduce an artificial field inhomogeneity along the *x*‐direction. This inhomogeneity is modeled correctly for the quasi‐continuous field map (B) but not for the conventional sequence (F). This propagates into the PDFF maps where the quasi‐continuous acquisition (D) remains consistent across all phantom vials, while the Cartesian six‐point sequence (H) exhibits a fat/water swap in the 75% vial (white arrow). These findings demonstrate the robustness of the proposed quasi‐continuous acquisition against appreciable *B*_0_ inhomogeneity compared with the conventional Cartesian method.

## Data Availability

The data that support the findings of this study are available on request from the corresponding author. The data are not publicly available due to privacy or ethical restrictions.
