# Supplementary material for: Fat/Water Separation at 7 T Using a 3D Radial Sequence With Quasi‐Continuous Echo Times
Source: Magn Reson Med. 2026 Feb 26;96(1):96–108. doi: 10.1002/mrm.70323 (PMC13156425; doi:10.1002/mrm.70323)
Supplement: Supplementary file 1 — Figure S1: Results of the measured gradient trajectory using the thin‐slice method and its impact on the images. (A–C) Measured gradient waveforms in comparison with the nominal gradient waveform for x‐, y‐, and z‐direction. Deviations are most pronounced at the beginning of the readout and immediately after the ramp‐up (zoomed‐in regions). The measured waveforms were incorporated into the reconstruction pipeline. (D–G) Water and fat images obtained with image data sets using the nominal gradient waveform (D and E) and the measured gradient waveform (F and G). Incorporating the measured trajectory substantially improves image quality for both contrast components. The blurred edges, particularly on the left side of the images, are effectively corrected, resulting in sharper depiction of the entire calf. Figure S2: Quantitative assessment of the off‐resonance correction on the fat images. (A) Fat image which was reconstructed at 0 ppm (without off‐resonance correction). (B) Fat image which was reconstructed at 3.5 ppm (with off‐resonance correction). In both images, a red horizontal line indicates the location of the intensity profiles shown in (C). The normalized line profiles demonstrate that the fat image with off‐resonance correction exhibits a much steeper signal drop, approaching zero between edges, whereas the uncorrected image retains elevated intensity levels, indicating blurring and signal spreading. This confirms that the off‐resonance correction improves the sharpness of the fat images. Figure S3: Phantom FWS results across three sagittal planes. The top row (A–C) shows the PDFF maps of the three sagittal slices, with the respective VFF ground truth values indicated inside the vials. The bottom row (D–F) displays the difference between the estimated PDFF maps and the ground truth. No large deviations appear in the sagittal plane, demonstrating the spatial robustness of the proposed method for the phantom data. The deviations that are visible are evenly dis [file MRM-96-96-s001.docx]

**Supplementary Material**

**
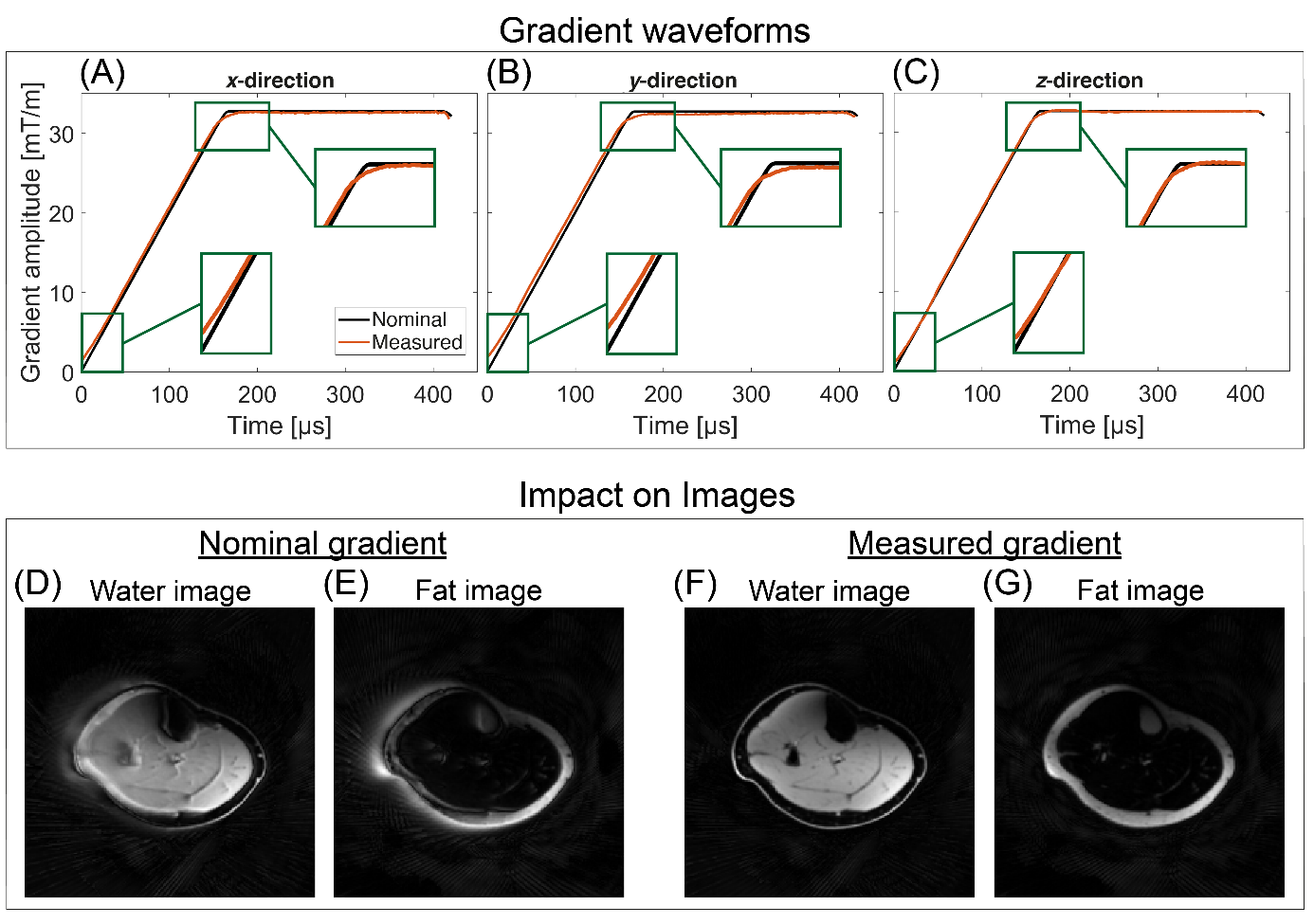
Figure S1:** Results of the measured gradient trajectory using the thin-slice method and its impact on the images. (A–C) Measured gradient waveforms in comparison with the nominal gradient waveform for *x*-, *y*-, and *z*-direction. Deviations are most pronounced at the beginning of the readout and immediately after the ramp-up (zoomed-in regions). The measured waveforms were incorporated into the reconstruction pipeline. (D–G) Water and fat images obtained with image data sets using the nominal gradient waveform (D & E) and the measured gradient waveform (F & G). Incorporating the measured trajectory substantially improves image quality for both contrast components. The blurred edges, particularly on the left side of the images, are effectively corrected, resulting in sharper depiction of the entire calf.


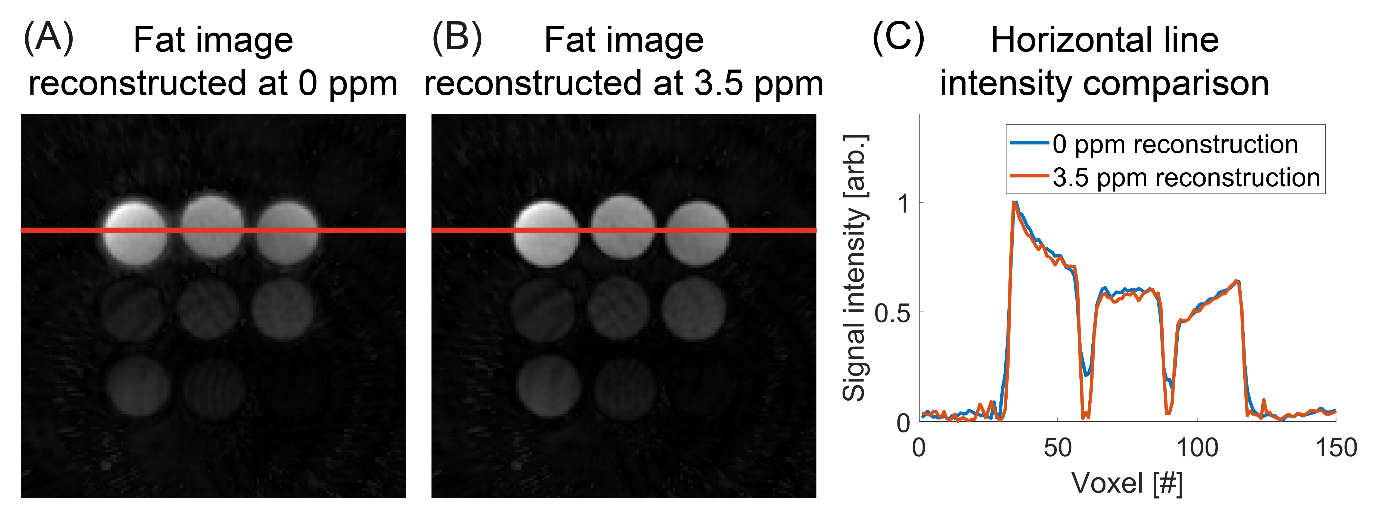
**Figure S2:** Quantitative assessment of the off-resonance correction on the fat images. (A) Fat image which was reconstructed at 0 ppm (without off-resonance correction). (B) Fat image which was reconstructed at 3.5 ppm (with off-resonance correction). In both images, a red horizontal line indicates the location of the intensity profiles shown in (C). The normalized line profiles demonstrate that the fat image with off-resonance correction exhibits a much steeper signal drop, approaching zero between edges, whereas the uncorrected image retains elevated intensity levels, indicating blurring and signal spreading. This confirms that the off-resonance correction improves the sharpness of the fat images.


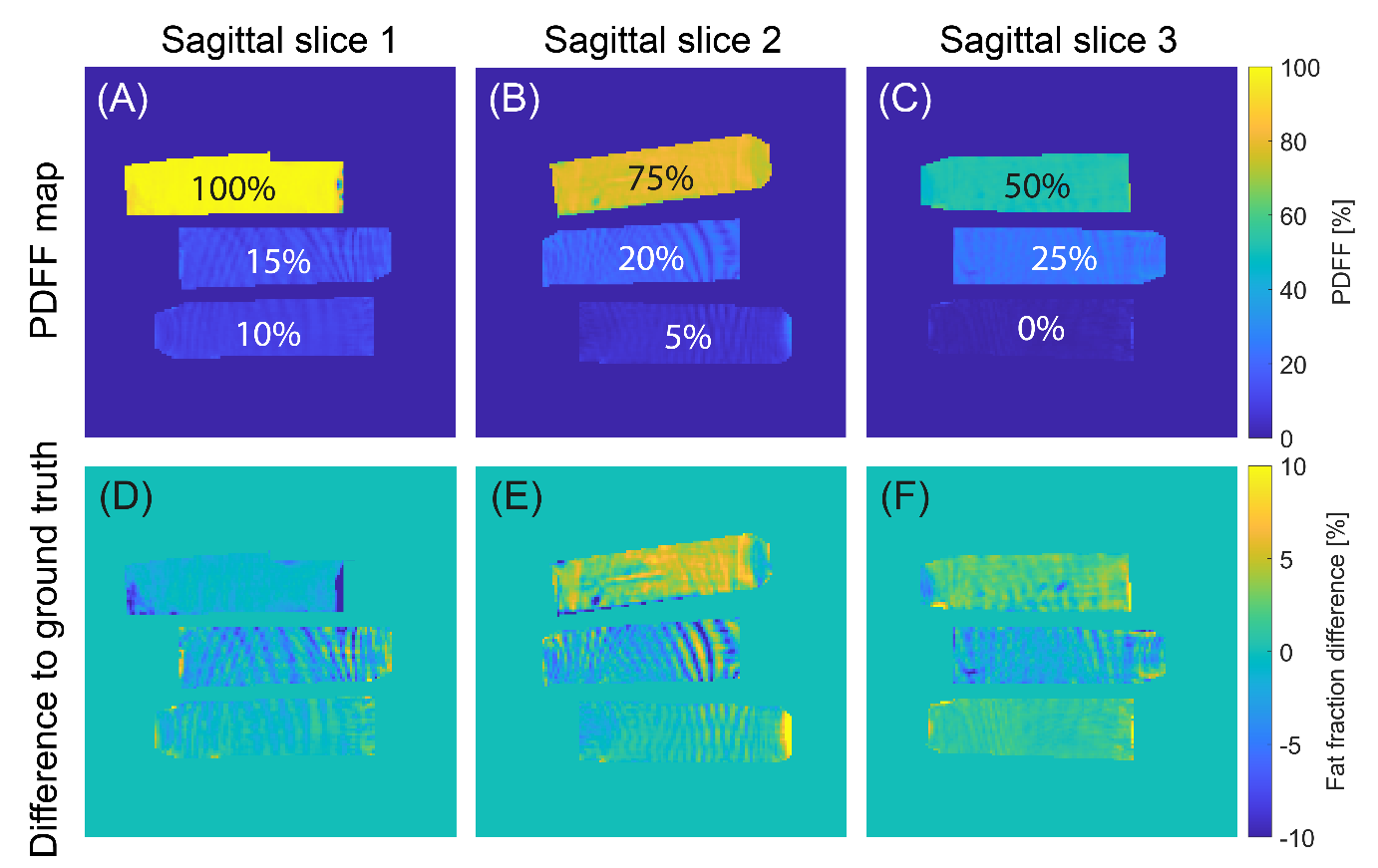


**Figure S3:** Phantom FWS results across three sagittal planes. The top row (A–C) shows the PDFF maps of the three sagittal slices, with the respective VFF ground truth values indicated inside the vials. The bottom row (D–F) displays the difference between the estimated PDFF maps and the ground truth. No large deviations appear in the sagittal plane, demonstrating the spatial robustness of the proposed method for the phantom data. The deviations that are visible are evenly distributed within the corresponding vials and are consistent with the transverse results shown in Figure 4. This spatial heterogeneity likely originates from temporally averaged phase evolution introduced by the sliding window reconstruction, which affect the resulting fat image and propagate into the PDFF maps.


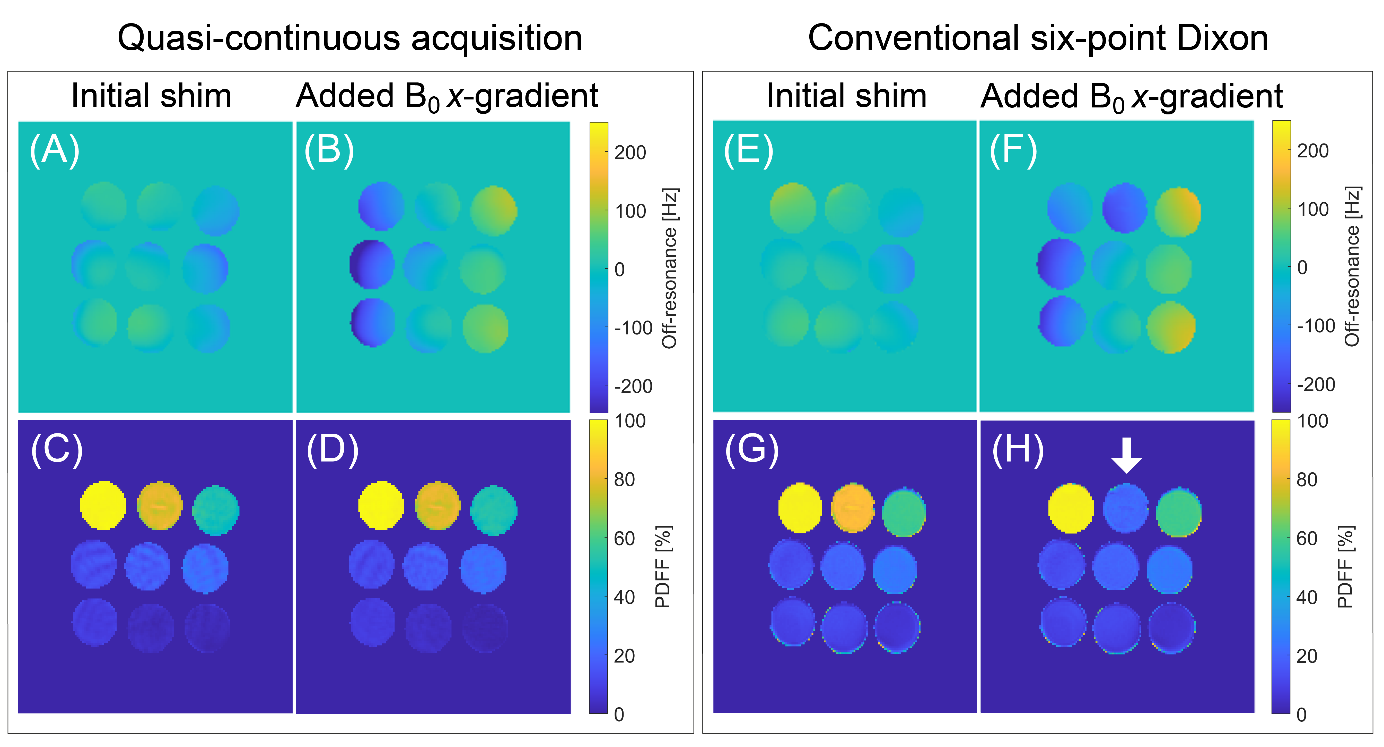


**Figure S4:** Phantom FWS results under two different shim conditions for both the quasi-continuous (A–D) and the conventional six-point Dixon sequences (E–H). The top row shows the B_0_ field maps and the bottom row shows the corresponding PDFF maps. The columns labeled “initial shim” display the results after a standard shimming protocol. As expected, the field maps (A & E) exhibit no substantial off-resonances, and the PDFF estimates (C & G) agree well with the ground truth values. The columns labeled “added B_0_ *x*-gradient” show the results after manually adjusting the shim currents to introduce an artificial field inhomogeneity along the *x*-direction. This inhomogeneity is modeled correctly for the quasi-continuous field map (B) but not for the conventional sequence (F). This propagates into the PDFF maps where the quasi-continuous acquisition (D) remains consistent across all phantom vials, while the Cartesian six-point sequence (H) exhibits a fat/water swap in the 75% vial (white arrow). These findings demonstrate the robustness of the proposed quasi-continuous acquisition against appreciable B_0_ inhomogeneity compared with the conventional Cartesian method.
